# Farnesoid X receptor promotes non-small cell lung cancer metastasis by activating Jak2/STAT3 signaling via transactivation of IL-6ST and IL-6 genes

**DOI:** 10.1038/s41419-024-06495-y

**Published:** 2024-02-15

**Authors:** Xiuye Jin, Bin Shang, Junren Wang, Jian Sun, Jing Li, Bin Liang, Xingguang Wang, Lili Su, Wenjie You, Shujuan Jiang

**Affiliations:** 1 Department of Respiratory and Critical Care Medicine, Shandong Provincial Hospital affiliated to Shandong First Medical University, Jinan, Shandong 250021 China; 2 Department of Respiratory and Critical Care Medicine, Shandong Provincial Hospital, Shandong University, Jinan, Shandong 250021 China; 3 Shandong Key Laboratory of Infectious Respiratory Disease, Jinan, Shandong 250021 China; 4 Medical Science and Technology Innovation Center, Shandong First Medical University & Shandong Academy of Medical Sciences, Jinan, Shandong 250000 China; 5 Department of Respiratory and Critical Care Medicine, Xi’an Chest Hospital, Shanxi, 710100 China; 6 Department of Thoracic Surgery, Shandong Provincial Hospital affiliated to Shandong First Medical University, Jinan, Shandong 250021 China; 7 Department of Thoracic Surgery, Shandong Provincial Hospital, Shandong University, Jinan, Shandong 250021 China; 8 Department of Respiratory and Critical Care Medicine, Shandong Provincial Public Health Clinical Center, Jinan, Shandong 250013 China; 9 Department of Thoracic Surgery, Fudan University Shanghai Cancer Center, Shanghai, 200032 China

**Keywords:** Metastasis, Non-small-cell lung cancer

## Abstract

Metastasis accounts for the majority of cases of cancer recurrence and death in patients with advanced non-small cell lung cancer (NSCLC). Farnesoid X Receptor (FXR) is a bile acid nuclear receptor that was recently found to be upregulated in NSCLC tissues. However, whether and how FXR regulates NSCLC metastasis remains unclear. In the present study, it was found that FXR promoted the migration, invasion, and angiogenic ability of NSCLC cells in vitro, and increased NSCLC metastasis in a mouse model in vivo. Mechanistic investigation demonstrated that FXR specifically bound to the promoters of *IL-6ST* and *IL-6* genes to upregulate their transcription, thereby leading to activation of the Jak2/STAT3 signaling pathway, which facilitated tumor migration, invasion, and angiogenesis in NSCLC. Notably, Z-guggulsterone, a natural FXR inhibitor, significantly reduced FXR^high^ NSCLC metastasis, and decreased the expression of FXR, IL-6, IL-6ST, and p-STAT3 in the mouse model. Clinical analysis verified that FXR was positively correlated with IL-6, IL-6ST and p-STAT3 expression in NSCLC patients, and was indicative of a poor prognosis. Collectively, these results highlight a novel FXR-induced IL-6/IL-6ST/Jak2/STAT3 axis in NSCLC metastasis, and a promising therapeutic means for treating FXR^high^ metastatic NSCLC.

## Introduction

Non-small cell lung cancer (NSCLC), which accounts for ~85% of all new lung cancer cases, represents the leading cause of cancer-related death worldwide [1]. Despite recent therapeutic advances, the clinical outcomes for NSCLC patients remain poor, with a 5-year survival rate of only 18%, which can be largely attributed to metastatic spread [2]. It is well acknowledged that the invasion-metastasis cascade primarily involves local migration and invasion, intravasation, survival in circulation, extravasation, and colonization in distant organs [3]. In addition, the primary tumors can also stimulate the formation of new blood vessels, termed neo-angiogenesis, to assist tumor metastasis [4]. Nevertheless, to date, our knowledge regarding the biological underpinnings that underlie the metastatic process is still lacking.

Signal transducer and activator of transcription-3 (STAT3) is a member of the STAT family of cytoplasmic transcription factors that regulates gene expression and is involved in cell proliferation, differentiation, anti-apoptosis, angiogenesis, metastasis, and immune responses [5, 6]. When activated, STAT3 translocates to the nucleus and binds to specific DNA sequences as a homodimer to induce target gene transcription. As a canonical stimulus, the cytokine IL-6 binds to its ɑ-receptor subunit, IL-6Rɑ, triggering an activated signaling function of another receptor subunit, IL-6ST, also known as gp130, which recruits and elicits the phosphorylation of Janus kinases-1 (Jak1) and Jak2. The activated Jak2 in turn phosphorylates the cytoplasmic tyrosine residues on IL-6 receptors, which act as a dock for the SH2 domain of STAT3, resulting in the phosphorylation (at residue of Tyr705) and activation of STAT3 homodimer [7]. Aberrant activation of STAT3 signals in tumor cells is tightly associated with tumor metastasis and angiogenesis [8]. Chang et al. reported that IL-6 expression is increased at the tumor-leading edge of invasive breast tumors [9]. IL-6 induces metastasis and angiogenesis and promotes myeloid cell recruitment in breast cancer via activation of an IL-6/Jak1/2/STAT3 signaling pathway [9]. Another study emphasized the pivotal role for IL-6/Jak2/STAT3 signaling pathway in carcinogen Bisphenol A-induced osteosarcoma metastasis both in vitro and in vivo [10]. Therefore, targeting the IL-6/Jak2/STAT3 pathway has recently emerged as an appealing approach for cancer therapy.

Farnesoid X Receptor (FXR) belongs to a nuclear receptor superfamily that is highly expressed in enterohepatic tissues [11]. Once activated by ligands, for example, bile acids (BAs), FXR translocates from the cytoplasm to the nucleus, and transcriptionally regulates downstream target genes related to bile acid, cholesterol, glucose, and lipid metabolism [12, 13]. Recently, FXR has been implicated either as an oncogene or as a tumoricidal gene in multiple malignancies, including liver, intestinal, breast, and pancreatic cancer [14–17]. We previously found that FXR expression is upregulated in NSCLC compared with peri-cancerous lung tissues, and that the levels of its ligands deoxycholic acid, ursodeoxycholic acid, and chenodeoxycholic acid are increased in the serum from patients with NSCLC compared with healthy controls [18, 19]. FXR promotes NSCLC cell proliferation via transcriptionally activating *CCND1* [18]. Additionally, an immunosuppressive role for FXR has been found in the tumor microenvironment, which would also facilitate local tumor growth in NSCLC [20]. However, as a multi-functional transcriptional factor, whether FXR contributes to NSCLC metastasis remains unclear.

In the present study, we demonstrated that FXR promotes the migration, invasion, and angiogenic ability of NSCLC cells in vitro, and increases NSCLC metastasis in mouse models in vivo. Importantly, we discovered a novel mechanism that FXR promotes NSCLC metastasis via binding specifically to the promoters of *IL-6ST* and *IL-6* to increase their transcription and activate the downstream Jak2/STAT3 signaling pathway. Clinically, FXR was confirmed to be positively correlated with IL-6, IL-6ST and phosphorylated STAT3 in NSCLC patients, which is indicative of a poor prognosis. Finally, the anti-metastatic activity of a FXR inhibitor against FXR^high^ NSCLC was verified in a mouse model in vivo. Our findings showed that FXR-induced IL-6/IL-6ST/Jak2/STAT3 signaling promoted NSCLC metastasis, highlighting a novel therapeutic option for treating advanced or metastatic NSCLC.

## Materials and methods

### Cell lines and reagents

Human NSCLC cell lines A549 and H1975, and human umbilical vein endothelial cells (HUVECs) were purchased from the American Type Culture Collection (ATCC). All cell lines used in this study were cultured in RPMI-1640 (Invitrogen; Thermo Fisher Scientific, Inc., MA) supplemented with 10% fetal bovine serum (FBS) and 1% penicillin-streptomycin. All cell lines were confirmed to be *Mycoplasma*-free, authenticated using short tandem repeat (STR) profiling, and passaged no more than 25 times after thawing. Z-guggulsterone (Z-GS;cat. no. sc-204414; Santa Cruz Biotechnology, Inc., CA) and STAT3 signaling pathway inhibitor, stattic (cat. no. abs812053; Absin Bioscience Inc., Shanghai, China) were endotoxins-free and dissolved in Dimethylsulphoxide (DMSO) according to the manufacturer’s instructions.

### Small interfering (si) RNA transfection

SiRNA transfections were performed using Lipofectamine^®^ 2000 (Invitrogen; Thermo Fisher Scientific, Inc.) according to the manufacturer’s instruction. The target sequences of the siRNAs used were: 5′-GGACCATGAAGACCAGATT-3′ (siRNA-1) and 5′-GACCTCGACAACAAAGTCA-3′ (siRNA-2) for human FXR knockdown, 5′-CCAACAATCCCAAGAATGT-3′ (siRNA-1) and 5′-GCAAAAAGTTTCCTACAAA-3′ (siRNA-2) for human STAT3 knockdown, and 5′-GGAACTGTCTAGTATCTTA-3′ (siRNA-1) and 5′-CCTCATGCACTGTTGATTA-3′ (siRNA-2) for human IL-6ST knockdown. The target sequence (5’-TTCTCCGAACGTGTCACGT-3’) was used as a negative control (NC; Shanghai GenePharma Co., Ltd, Shanghai, China).

### Lentiviral vectors and cell infection

Lentiviral vectors were generated and packaged by OBiO Biotechnology (Shanghai, China), using pCMV-NR1H4-PGK-PuroR plasmids, which carry the full-length human *NR1H4* (GenBank accession no. NM_005123.3), or pCMV-EGFP-2A-PuroR-U6-shRNA plasmids, which generate shRNA targeting FXR or NCshRNA as described above. Cell infection was performed using lentiviral vectors as described in our previous study [18].

### Patients and data collection

Between 2015 and 2020, primary NSCLC samples were consecutively collected from patients who underwent surgery at the Department of Thoracic Surgery in Shandong Provincial Hospital affiliated to Shandong First Medical University. The survival information was obtained via regular follow-up clinic visits or telephone calls. The inclusion criteria were as follows: 1. complete resection with curative intent; 2. pathologically confirmed NSCLC by two independent pathologists. The exclusion criteria were as follows: 1. treatment with neo-adjuvant chemotherapy or radiotherapy; 2. medical history of other malignant tumors. In total, 144 NSCLC samples were enrolled in the present study. This study was approved by the Ethics Committee of Shandong Provincial Hospital. Written informed consent was obtained from all enrolled patients. All experiments were conducted in accordance with the principles of the Declaration of Helsinki and the guidelines approved by the Ethics Committee in Shandong Provincial Hospital.

### Immunohistochemical (IHC) analysis

Paraffin-embedded clinical NSCLC samples and metastatic lung tumors in mouse models were subjected to IHC staining as previously described [21]. For the detection of FXR, IL-6, IL6ST and p-STAT3, the following primary antibodies were used: anti-bile acid receptor NR1H4 (1:100; cat. no. ab187735; Abcam, Cambridge, UK), anti-IL-6 (1:100; cat. no. ab9324; Abcam), anti-CD130 (gp130) (1:100; cat. no. ab227058; Abcam) and anti-Phospho-STAT3 (Tyr705) (1:200; cat. no. 9145s; Cell Signaling Technology, Inc., Beverly, MA). Concentration-matched non-specific mouse or rabbit IgG served as isotype controls. The IHC staining results were scored independently and blindly by two skilled pathologists, and a final consensus was reached. The staining intensity of tumor cells was scored as negative (0), weak (1), medium (2), and strong (3), respectively. The percentage of positive cells was scored as follows: 0% (0), 1%–25% (1), 26%–50% (2), 51%–75% (3), and 76%–100% (4), respectively. The final IHC scores of human FXR, IL-6, IL6ST and p-STAT3 were obtained by multiplying the staining intensity score with the positive-cell percentage score, and stratified as follows: Low, score < 6 or high, score ≥ 6, in line with our previous studies [18, 19].

### Transwell assay

Transwell assays were performed to evaluate the migratory and invasive abilities of NSCLC cells. Briefly, 3 × 10^4^ cells in medium without FBS were placed into the upper chamber of an insert (8-μm pore size). Medium containing 20% FBS was added to the lower chamber as the chemotactic factor. For the invasion assays, the Transwell inserts were pre-coated with 50 µl/well Matrigel (Corning, Inc., New York, NY). After incubation for 36 h (for migration assay) or 48 h (for invasion assay), cells that had not migrated or invaded through the pores were removed with cotton swabs. The Transwell chambers were fixed with 4% polyformaldehyde for 15 min and stained with 0.1% crystal violet solution for 20 min. A microscope was used for the quantification of the migrated and invaded cells from five random fields of view at a magnification of ×200.

### Angiogenesis assay

Briefly, 96-well plates were coated with 50 µl/well Matrigel (Corning, Inc.) and placed in a 37 °C humidified incubator for 1 h. HUVECs were exposed to the culture medium obtained from A549 or H1975 cells for 36 h, and then seeded in the coated 96-well plates (3 × 10^4^ cells/well). After 3 h, the newly formed tubes were imaged using a microscope at a magnification of ×100.

### ELISA

The amounts of IL-6 in the culture supernatant of A549 or H1975 cells were measured using a Human IL-6 Quantikine ELISA Kit (R&D Systems, Abingdon, UK) according to the manufacturer’s instructions.

### Western blot analysis

Western blotting was performed in accordance with our previously described procedures [18]. The following primary antibodies were diluted according to the manufacturers’ recommendations: anti-human bile acid receptor NR1H4 (cat. no. ab187735; Abcam), anti-IL-6 (cat. no. ab9324; Abcam), anti-CD130 (gp130) (cat. no. ab283685; Abcam), anti-phosphorylated (p)-Jak2 (Tyr1007/1008) (cat. no. 3771s; Cell Signaling Technology, Inc.), anti-Jak2 (cat. no. 3230s; Cell Signaling Technology, Inc.), anti-p-STAT3 (Tyr705) (cat. no. 9145s; Cell Signaling Technology, Inc.), anti-STAT3 (cat. no. 12640s; Cell Signaling Technology, Inc.), anti-IL-6Rα/CD126 (cat. no. 18935s; Cell Signaling Technology, Inc.), anti-YAP (cat. no. 12395s; Cell Signaling Technology, Inc.), anti-HES1 (cat. no. 11988s; Cell Signaling Technology, Inc.), anti-Cleaved Notch1 (cat. no. 4147s; Cell Signaling Technology, Inc.), anti-GAPDH (cat. no. 92310sf; Cell Signaling Technology, Inc.), and anti-p-YAP1 (Tyr357) (cat. no. Y0771; Sigma-Aldrich, Inc., USA).

### Quantitative Real-Time Polymerase Chain Reaction (qRT-PCR)

Total RNA was extracted from cells using a TRIzol^®^ reagent (Invitrogen; Thermo Fisher Scientific, Inc.). According to the manufacturer’s protocol, aliquots of 1 μg RNA were used as a template for reverse transcription to obtain cDNA. qRT-PCR analysis was performed using ChamQ Universal SYBR qPCR Master Mix (Vazyme Biotech Co., Ltd, Nanjing, China) on a Roche light cycle 480 system (Roche Diagnostics, Basel, Switzerland). The primer sequences used were: Human IL-6 forward, 5’-CAATGAGGAGACTTGCCTGGT-3’ and reverse, 5’-GCAGGAACTGGATCAGGACT-3’; IL-6ST forward, 5’-CCATAGTCGTGCCTGTTTGC-3’ and reverse, 5’-CTTGGAGGAGTGTGAGGTGAC-3’; IL-6Rα forward, 5’-GCAGTGTGTGTAGAGAGCCG-3’ and reverse, 5’-CAGAGGCGGACAGGCTAATG-3’; and β-actin forward, 5′-TTGCTGATCCACATCTGCT-3′ and reverse, 5′-GACAGGATGCAGAAGGAGAT-3′.

### Chromatin immunoprecipitation (ChIP) assay

ChIP assay was performed using a SimpleChIP Plus Enzymatic Chromatin IP Kit (Cell Signaling Technology, Inc.) according to the manufacturer’s instruction, as described in our previous study [18]. ChIP-qPCR primers for human *IL-6ST* promoter were : Primer 1 forward, 5’-TTGCCCAAGGTCACAGAGATG-3’ and reverse, 5’-TGCTGAGATAACAGGTGTGATGC-3’; Primer 2 forward, 5’-CGATCTATGTTTTACTCTATCTGAATCC-3’ and reverse, 5’-AAAGTAAACACCCAAAGGTAGAGG-3’; and Primer 3 forward, 5’-CCAAGGGAGCTAGGCGGTC-3’ and reverse, 5’-GGCGGAAAGAGCGGAATGT-3’. ChIP-qPCR primers for human *IL-6* promoter were: Primer 1 forward, 5’-GAGGACCACCGTCTCTGTTTAG-3’ and reverse, 5’-GTGACCTCTGTTGGGCATTTAC-3’; Primer 2 forward, 5’-AGCACAAGGCAAACCTCTGG-3’ and reverse, 5’-CCTGTGAGCGGCTGTTGTAG-3’; and Primer 3 forward, 5’-GCTGCGATGGAGTCAGAGGA-3’ and reverse, 5’-ACTCAGCACTTTGGCATGTCTT-3’; Primer 4 forward, 5’-CAAGACATGCCAAAGTGCTGA-3’ and reverse, 5’-TCTTTGTTGGAGGGTGAGGGT-3’.

### Luciferase reporter assay

Luciferase reporter plasmids carrying the wild-type *IL-6ST* promoter, the second putative FXRE motif-deleted *IL-6ST* promoter, the wild-type *IL-6* promoter, and the first-to-third putative FXRE motifs-deleted *IL-6* promoter were generated by Shanghai GenePharma Co., Ltd. Luciferase reporter assays were performed using a Dual-Luciferase Reporter Assay System (Promega Corp., Madison, WI) in accordance with our previously described procedures [18].

### Animal experiments

Five-week-old BALB/c female nude mice were housed and maintained under special pathogen-free (SPF) conditions, and randomly (random number grouping method) allocated to control and experimental groups (*n* = 6 per group). FXR-silenced or NC H1975 stable cells, or FXR-overexpressed or mock A549 stable cells (3 × 10^6^ cells per mouse, in 200 µl PBS) were injected into the tail vein of mice. For in vivo FXR inhibition study, mice harboring A549 cells with or without stable FXR overexpression were randomly grouped and treated intraperitoneally (i.p.) with vehicle or Z-GS (10, 20, and 40 mg/kg) every 3 days. A total of 55 days after tumor cell inoculation, mouse lungs were surgically excised for imaging, and then subjected to H&E staining and IHC staining analysis. The animal experiments were approved by the Institutional Animal Care and Use Committee in Shandong Provincial Hospital. All animal procedures complied with the guidelines of the Institutional Animal Care and Use Committee in Shandong Provincial Hospital.

### Statistical analysis

The sample size in each experiment was chosen to ensure adequate power to detect a pre-specified effect. Statistical analysis was performed using SPSS version 26.0 (IBM Corp., Armonk, NY) and GraphPad Prism version 8.0 (GraphPad Software, Inc., San Diego, CA). Quantitative data are presented as the mean ± standard deviation (SD), and assessed for normal distribution via a Kolmogorov-Smirnov test. The two groups were compared using a Mann-Whitney *U* test and a Student’s *t* test for skewed distributed data or non-skewed distributed data, respectively. The variance was similar between the groups that were statistically compared. A Spearman’s rank correlation test was conducted to analyze the correlation between FXR and p-STAT3 (Tyr705), between FXR and IL-6ST, and between FXR and IL-6 in the clinical NSCLC cohort, between *NR1H4* and *STAT3* mRNA, between *NR1H4* and *IL-6ST* mRNA, and between *NR1H4* and *IL-6* mRNA in NSCLC from The Cancer Genome Atlas (TCGA) cohort. The TCGA accession codes were listed in Supplementary Table S1. All tests were two-sided. *p* < 0.05 was considered to indicate a statistically significant difference.

## Results

### FXR promotes migration, invasion, and angiogenic ability of NSCLC cells

**Fig. 1:**
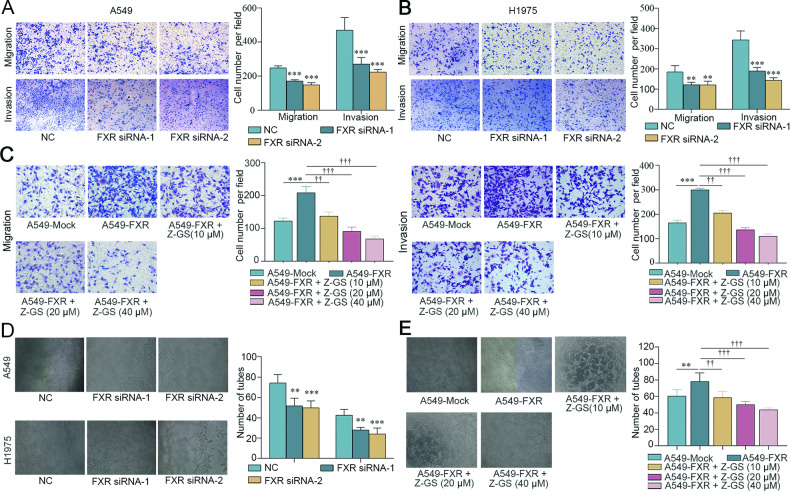
FXR promotes NSCLC cell migration, invasion, and angiogenesis. A549 and H1975 cells were transfected with NC- or FXR-siRNAs for 48 h. The migratory and invasive abilities of A549 (**A**) and H1975 cells (**B**) were analyzed by Transwell migration and invasion assays. **C** A549 cells were infected with mock or FXR-overexpressed lentiviral vectors to establish stable cell lines, followed by treatment with increasing concentrations of Z-GS (0, 10, 20, and 40 µM) for 48 h. Cell migratory (left panels) and invasive (right panels) abilities were assessed by Transwell assays. The angiogenic abilities of A549 and H1975 cells transfected with NC- or FXR-siRNAs (**D**) and FXR-overexpressed A549 stable cells treated with increasing concentrations of Z-GS (0, 10, 20, and 40 µM) (**E**) were evaluated by angiogenesis assays. Representative images (magnification, ×200) and quantitative results are shown. Data represents mean ± SD from at least three independent experiments. **p < 0.01, *** *p* < 0.001, compared with the NC or mock group. ^††^*p* < 0.01, ^†††^*p* < 0.001, compared with A549-FXR group.

First, we attempted to evaluate the function of FXR in NSCLC metastasis in vitro. Two effective siRNAs were used to knock down FXR in A549 and H1975 cells. Lentiviral vectors were used to overexpress FXR in A549 cells, which were then treated with Z-GS, a widely recognized FXR inhibitor [22]. In Transwell assays, FXR silencing significantly decreased the migratory and invasive efficiency of A549 and H1975 cells (Fig. 1A, B). By contrast, FXR overexpression resulted in increased migration and invasion cell numbers in A549 cells, which was reversed with increasing concentrations of Z-GS (Fig. 1C).

Since angiogenesis is necessary for tumor invasion and metastasis [4], the effect of FXR on the angiogenic ability of NSCLC cells was examined. FXR-silenced or -overexpressed NSCLC cells were cocultured with HUVECs at a 1:5 ratio, respectively. Results showed that FXR silencing in A549 or H1975 significantly minimized the number of newly formed tubes in the cocultured HUVECs (Fig. 1D). In comparison, overexpression of FXR in A549 led to an increased tube formation in the cocultured HUVECs, which was abrogated by Z-GS in a dose-dependent manner (Fig. 1E). Collectively, these findings demonstrated that FXR promotes the migration, invasion, and angiogenic ability of NSCLC cells in vitro.

### The effect of FXR on NSCLC migration, invasion, and angiogenesis is dependent on the Jak2/STAT3 signaling pathway

**Fig. 2:**
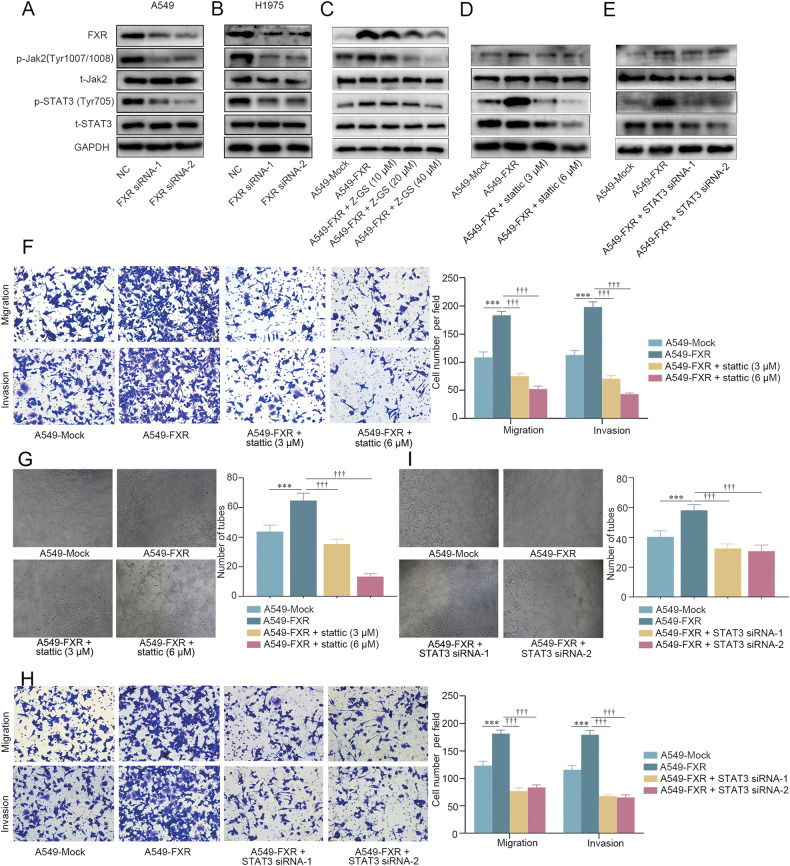
The promoting effect of FXR on NSCLC migration, invasion, and angiogenesis is dependent on the Jak2/STAT3 signaling pathway. Expression levels of FXR, p-Jak2 (Tyr1007/1008), t-Jak2, p-STAT3 (Tyr705), and t-STAT3 protein in FXR-silenced A549 cells (**A**), FXR-silenced H1975 cells (**B**), and FXR-overexpressed A549 stable cells treated with increasing concentrations of Z-GS (0, 10, 20, and 40 µM) (**C**) were examined by Western blotting. GAPDH was used as a loading control. **D**–**I** FXR-overexpressed A549 stable cells were treated with increasing concentrations of Stattic (0, 3, and 6 µM), or transfected with NC- or STAT3-siRNAs for 48 h. Expression levels of p-Jak2 (Tyr1007/1008), t-Jak2, p-STAT3 (Tyr705), and t-STAT3 protein in FXR-overexpressed A549 stable cells treated with Stattic (**D**) or transfected with STAT3-siRNAs (**E**) were examined by Western blotting. The migratory and invasive abilities of FXR-overexpressed A549 stable cells treated with Stattic (**F**) or transfected with STAT3-siRNAs (**H**) were determined by Transwell assays. The angiogenic abilities of FXR-overexpressed A549 stable cells treated with Stattic (**G**) or transfected with STAT3-siRNAs (**I**) were examined by angiogenesis assays. For (**F**–**I**), representative images (magnification, ×200) and quantitative results are shown. Data are presented as mean ± SD from at least three independent experiments. ***p < 0.001, compared with the mock group. ^†††^*p* < 0.001, compared with A549-FXR group.

The intracellular signaling pathways by which FXR promotes NSCLC migration, invasion, and angiogenesis were further explored. Previous studies have documented that the Jak2/STAT3 signaling pathway is associated with tumor metastasis and angiogenesis [8–10]. We thus hypothesized that the Jak2/STAT3 pathway may be involved in the regulation of NSCLC migration, invasion, and angiogenesis by FXR. Herein, our results indicated that the phosphorylation of both Jak2 (Tyr1007/1008) and STAT3 (Tyr705) was significantly downregulated in A549 and H1975 cells with FXR knockdown, whereas the levels of total Jak2 and STAT3 remained unchanged (Fig. 2A, B). Conversely, A549 cells overexpressing FXR exhibited elevated levels of phosphorylated Jak2 (Tyr1007/1008) and STAT3 (Tyr705), which were reversed with increasing doses of Z-GS (Fig. 2C). These findings revealed that FXR activates the Jak2/STAT3 signaling pathway in NSCLC cells.

To determine whether the Jak2/STAT3 signaling pathway is involved in FXR-induced NSCLC migration, invasion, and angiogenesis, stably FXR-overexpressing A549 cells were treated with STAT3 inhibitor stattic or transfected with STAT3-siRNAs. Western blotting confirmed the increased phosphorylation of STAT3 (Tyr705) in FXR-overexpressed A549 cells, which was markedly reduced upon treatment with either stattic (3 or 6 µM) or STAT3-siRNAs (Fig. 2D, E). Subsequent functional experiments showed that the STAT3 inhibitor significantly reversed the promoting effect of FXR on migration, invasion, and angiogenic ability in A549 cells (Fig. 2F, G). Meanwhile, STAT3 knockdown significantly reduced the increase in migration, invasion, and angiogenesis efficiency in FXR-overexpressed A549 cells (Fig. 2H, I). Together, the above results suggested that FXR promotes migration, invasion, and angiogenesis by activating the Jak2/STAT3 signaling pathway in NSCLC cells.

### FXR promotes Jak2/STAT3-mediated tumor metastasis and angiogenesis through upregulation of IL-6 and IL-6ST in NSCLC

**Fig. 3:**
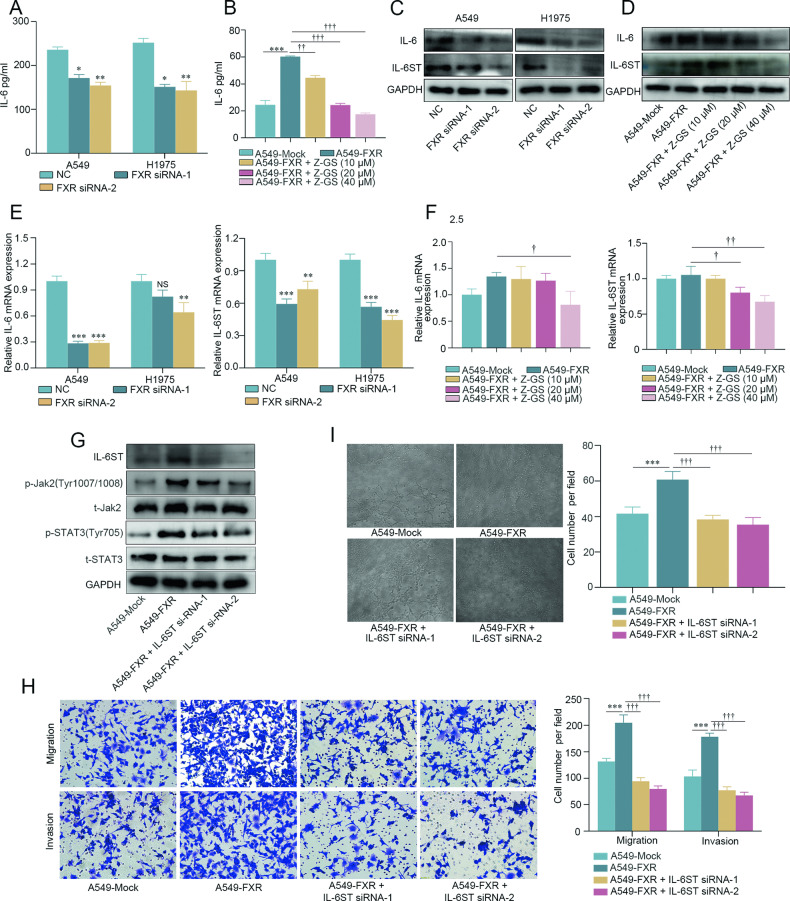
FXR promotes Jak2/STAT3-mediated NSCLC metastasis and angiogenesis by upregulating IL-6 and IL-6ST. ELISA showing the concentrations of IL-6 in the culture supernatant of FXR-silenced A549 and H1975 cells (**A**) and FXR-overexpressed A549 stable cells treated with increasing concentrations of Z-GS (0, 10, 20, and 40 µM) (**B**). Western blotting was performed to evaluate the protein levels of IL-6 and IL-6ST in FXR-silenced A549 and H1975 cells (**C**) and FXR-overexpressed A549 stable cells treated with increasing concentrations of Z-GS (0, 10, 20, and 40 µM) (**D**). GAPDH was used as a loading control. qRT-PCR was performed to examine the mRNA levels of IL-6 and IL-6ST in FXR-silenced A549 and H1975 cells (**E**) and FXR-overexpressed A549 stable cells treated with increasing concentrations of Z-GS (0, 10, 20, and 40 µM) (**F**). β-actin served as an internal control. **G**–**I** FXR-overexpressed A549 stable cells were transfected with NC- or IL-6ST-siRNAs for 48 h. **G** Expression levels of IL-6ST, p-Jak2 (Tyr1007/1008), t-Jak2, p-STAT3 (Tyr705), and t-STAT3 protein were examined by Western blotting. **H** Cell migratory and invasive abilities were assessed by Transwell assays. **I** Cell angiogenic abilities were evaluated by angiogenesis assays. For (**H**, **I**), representative images (magnification, ×200) and quantitative results are shown. Data represents mean ± SD from at least three independent experiments. *p < 0.05, **p < 0.01, ***p < 0.001, compared with the NC or mock group. ^†^*p* < 0.05, ^††^*p* < 0.01, ^†††^*p* < 0.001, compared with A549-FXR group. NS, not significant.

An in-depth analysis was performed to investigate the molecular basis underlying FXR-modulated Jak2/STAT3 activation in NSCLC. Since IL-6 is a well-known canonical activator of the Jak2/STAT3 signaling pathway that enhances NSCLC progression [7, 23], the upstream genes controlling Jak2/STAT3 signals, namely IL-6, IL-6ST, and IL-6Rɑ, were analyzed in NSCLC cells with FXR overexpression or knockdown. As shown in Fig. 3A, FXR silencing led to a significantly reduced level of IL-6 in the culture supernatant of either A549 or H1975 cells. By contrast, FXR overexpression significantly increased the production of IL-6 in A549 cells, which was dose-dependently reversed by Z-GS (Fig. 3B). In parallel, western blotting showed that FXR knockdown decreased the protein levels of IL-6 and IL-6ST, but increased IL-6Rɑ expression in both A549 and H1975 cells (Fig. 3C and Supplementary Fig. S1A). Conversely, FXR overexpression resulted in increased protein levels of IL-6 and IL-6ST in A549 cells, which were restrained by Z-GS in a dose-dependent manner (Fig. 3D). However, there was no obvious change in IL-6Rɑ protein expression in FXR-overexpressed A549 cells upon Z-GS treatment (Supplementary Fig. S1B). The mRNA levels of IL-6, IL-6ST, and IL6Rɑ were also examined. Results showed that following FXR knockdown, IL-6 and IL-6ST mRNA expression were significantly decreased either in A549 or in H1975 cells, whereas IL6Rɑ mRNA was excluded from this reduction (Fig. 3E and Supplementary Fig. S1C). On the other hand, A non-significant increase in IL-6 and IL-6ST mRNA expression was observed in FXR-overexpressed A549 cells, which was reduced by Z-GS in a dose-dependent manner (Fig. 3F). Meanwhile, an opposite change of IL-6Rɑ mRNA was observed in Z-GS-treated FXR-overexpressed A549 cells (Supplementary Fig. S1D). These data revealed that FXR upregulates IL-6 and IL-6ST, rather than IL6Rɑ, in NSCLC cells.

To determine the functional relevance of IL-6 and IL-6ST in FXR-modulated Jak2/STAT3 activation and tumor metastasis, stably FXR-overexpressing A549 cells were transfected with IL-6ST-siRNAs. Western blotting confirmed the efficiency of IL-6ST knockdown in FXR-overexpressed A549 cells (Fig. 3G). As shown in Fig. 3G, IL-6ST silencing significantly decreased the phosphorylation of Jak2 (Tyr1007/1008) and STAT3 (Tyr705) induced by FXR overexpression in stable A549 cells, indicating the inhibition of the Jak2/STAT3 signaling pathway. Additionally, the results of the Transwell and tube formation assays showed that the promoting effect of FXR overexpression on tumor migration, invasion, and angiogenic ability was almost completely abolished by IL-6ST knockdown in A549 cells (Fig. 3H, I). Taken together, the above results demonstrated that FXR promotes Jak2/STAT3-mediated tumor metastasis and angiogenesis through upregulation of IL-6 and IL-6ST in NSCLC cells.

Since the concomitant activation of STAT3, Src, YAP, and Notch were reported in IL-6ST-activated intestinal epithelial cells [24], we evaluated the levels of phosphorylated YAP1 (Tyr357), YAP, cleaved Notch1, and HES1 in FXR-silenced or -overexpressed NSCLC cells. However, though FXR activated the Jak2/STAT3 signaling pathway via upregulating IL-6 and IL-6ST, FXR silencing in A549 and H1975 significantly increased the levels of phosphorylated YAP1 (Tyr357), cleaved Notch1, and HES1 (Supplementary Fig. S1E), whereas FXR overexpression resulted in the inactivation of both YAP and Notch signaling pathways in A549 cells (Supplementary Fig. S1F).

### FXR transcriptionally upregulates IL-6ST and IL-6 via directly binding to the promoter

**Fig. 4:**
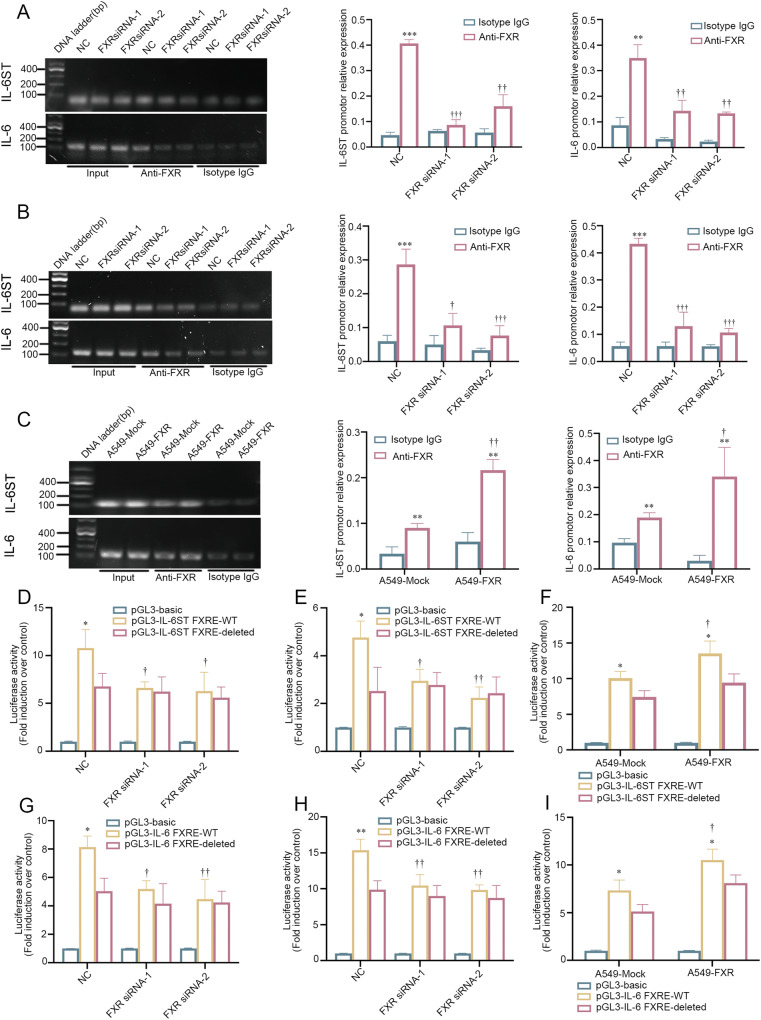
FXR directly activates *IL-6ST* and *IL-6* transcription in NSCLC cells. ChIP assays were performed in FXR-silenced A549 cells (**A**), FXR-silenced H1975 cells (**B**), and FXR-overexpressed A549 stable cells (**C**) using anti-human FXR/NR1H4 antibody and primer corresponding to the second putative FXRE motif in *IL-6ST* promoter, or primer corresponding to the first-to-third putative FXRE motifs in *IL-6* promoter. Representative PCR amplification products are shown (left bands). Enrichments of FXR in the putative FXRE motifs of *IL-6ST* and *IL-6* promoters were examined relative to input samples (right graphs). Chromatin obtained with isotype IgG and non-immunoprecipitated samples (input) served as negative and positive controls, respectively. FXR-silenced A549 cells (**D**), FXR-silenced H1975 cells (**E**), and FXR-overexpressed A549 stable cells (**F**) were transfected with a wild-type *IL-6ST* promoter plasmid (pGL3-IL-6ST FXRE-WT) or the second putative FXRE motif-deleted *IL-6ST* promoter plasmid (pGL3-IL-6ST FXRE-deleted) for 24 h. The luciferase activities were measured. FXR-silenced A549 cells (**G**), FXR-silenced H1975 cells (**H**), and FXR-overexpressed A549 stable cells (**I**) were transfected with a wild-type *IL-6* promoter plasmid (pGL3-IL-6 FXRE-WT) or the first-to-third putative FXRE motifs-deleted *IL-6* promoter plasmid (pGL3-IL-6 FXRE-deleted) for 24 h. The luciferase activities were measured. For (**D**–**I**), pGL3-basic plasmid served as the negative control. Data are shown as mean ± SD from at least three independent experiments. *p < 0.05, **p < 0.01, ***p < 0.001, compared with the isotype IgG (**A**–**C**), the second putative FXRE motif-deleted *IL-6ST* promoter plasmid (**D**-**F**), or the first-to-third putative FXRE motifs-deleted *IL-6* promoter plasmid (**G**–**I**). ^†^*p* < 0.05, ^††^*p* < 0.01, ^†††^*p* < 0.001, compared with the NC or mock group.

Next, we sought to characterize the detailed mechanisms responsible for FXR-induced IL-6 and IL-6ST upregulation in NSCLC cells. FXR functions as a transcription factor via binding to the FXR-responsive elements (FXREs) in the promoter regions of target genes either as an FXR-RXRɑ heterodimer or as a homodimer [25]. Gene sequence analysis using JASPAR (http://jaspar.genereg.net/) indicated that three potential FXREs (TCTGTGACCTT at nucleotide −1688/−1678, AAGTTCATTAAACCA at nucleotide −973/−959, and TTCATGACCCC at nucleotide −358/−348) were identified within 2000 bp upstream of the human *IL-6ST* transcription start site. Our ChIP assays revealed a site-specific binding of FXR to the second (AAGTTCATTAAACCA), rather than the first (TCTGTGACCTT) or third (TTCATGACCCC) putative FXRE sequence, in human *IL-6ST* promoter both in A549 and in H1975 cells (Supplementary Fig. S2A). Notably, FXR silencing in A549 and H1975 led to decreased binding of FXR to the second putative FXRE motif in the *IL-6ST* promoter region (Fig. 4A, B). In contrast, A549 cells with FXR overexpression resulted in significantly increased recruitment of FXR to this site of *IL-6ST* promoter, compared with mock A549 cells (Fig. 4C). To determine whether the binding of FXR to this FXRE sequence affected *IL-6ST* transcription, luciferase reporter assays were performed using plasmids carrying the *IL-6ST* promoter with or without the second FXRE motif. We observed that the wild-type *IL-6ST* promoter activity was significantly higher than that of the FXRE-deleted *IL-6ST* promoter plasmid both in A549 and in H1975 cells (Fig. 4D, E). Importantly, the knockdown of FXR in A549 and H1975 significantly reduced the activity of the wild-type *IL-6ST* promoter plasmid but had no effect on the activity of FXRE-deleted *IL-6ST* promoter plasmid (Fig. 4D, E). Conversely, overexpression of FXR in A549 resulted in increased luciferase activity of the wild-type *IL-6ST* promoter plasmid, while the FXRE-deleted *IL-6ST* promoter was excluded from this enhancement (Fig. 4F). These findings demonstrated that FXR activates IL-6ST transcription via direct binding to the second FXRE in the *IL-6ST* promoter in NSCLC cells.

Then, whether FXR transcriptionally regulated IL-6 in NSCLC was addressed. Gene sequence analysis showed that the −2000/+82 region of the human *IL-6* promoter contains six putative FXRE sequences (TGGATGACCTC at nucleotide −1456/−1446, TAAATGCCCAA at nucleotide −1393/−1383, GAGGTCACTGT at nucleotide −1380/−1370, GGGGTCACTTG at nucleotide −746/−736, TCTGGTCACTGA at nucleotide −405/−394, and TCAATGACGAC at nucleotide −219/−209). However, due to the location proximity, only one pair of ChIP-qPCR primers was designed to amplify DNA fragments containing the first-to-third putative FXRE motifs (TGGATGACCTC, −1456/−1446; TAAATGCCCAA, −1393/−1383; GAGGTCACTGT, −1380/−1370) in the *IL-6* promoter. ChIP assays demonstrated that FXR specifically interacted with the first-to-third, rather than the fourth (GGGGTCACTTG), fifth (TCTGGTCACTGA), or sixth (TCAATGACGAC) putative FXRE sequences, in the human *IL-6* promoter region both in the A549 and in H1975 cells (Supplementary Fig. S2B). Additionally, we found that FXR knockdown in A549 and H1975 cells significantly abolished the binding effect of FXR to the first-to-third putative FXRE motifs, while FXR overexpression in A549 cells apparently increased the recruitment of FXR to this fragment in the *IL-6* promoter (Fig. 4A–C). Luciferase reporter assays were conducted to verify the involvement of FXR in *IL-6* transcription. Our results showed that the luciferase activity of wild-type *IL-6* promoter plasmid was significantly higher than that of the first-to-third putative FXRE motifs-deleted *IL-6* promoter both in A549 and in H1975 cells (Fig. 4G, H). Notably, FXR silencing in A549 and H1975 cells led to a significant reduction in the activity of the wild-type *IL-6* promoter plasmids, rather than the first-to-third putative FXRE motifs-deleted *IL-6* promoter (Fig. 4G, H). In contrast, FXR overexpression in A549 cells significantly increased the transcription of the wild-type *IL-6ST* promoter (Fig. 4I). However, the luciferase activity of the first-to-third putative FXRE motifs-deleted *IL-6* promoter plasmid remained unchanged in FXR-overexpressed A549 cells (Fig. 4I). In combination, these results suggested that FXR activates IL-6 transcription via binding to the first-to-third putative FXRE sequences in the *IL-6* promoter in NSCLC cells.

### FXR inhibitor reduces FXR^high^ NSCLC metastasis in a mouse model

**Fig. 5:**
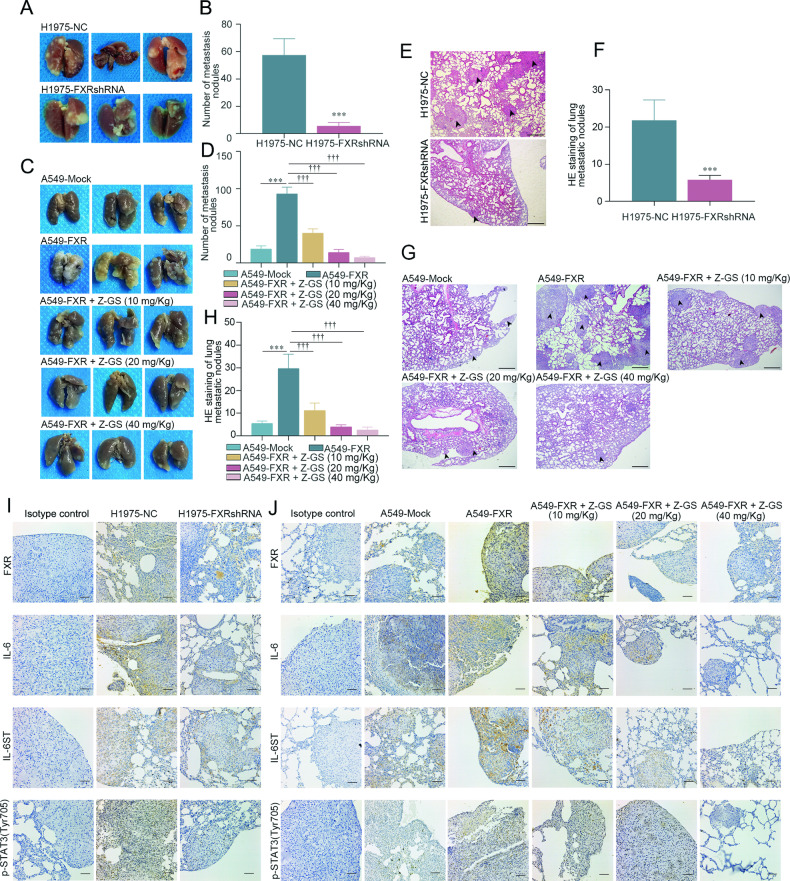
FXR increases NSCLC metastasis in vivo, which is reduced by FXR inhibitor Z-GS. **A**, **B** H1975 cells were infected with lentiviral vectors carrying NC- or FXR-shRNA to establish stable cell lines. Then, nude mice were inoculated with FXR-silenced H1975 stable cells by tail vein injection (3 × 10^6^ cells per mouse). After 55 days, the gross metastatic nodules on the mouse lung surface were photographed (**A**) and counted (**B**). **C**, **D** Nude mice were inoculated with FXR-overexpressed A549 stable cells by tail vein injection (3 × 10^6^ cells per mouse), and injected i.p. with increasing doses of Z-GS (0, 10, 20, and 40 mg/kg) every 3 days. After 55 days, the gross metastatic nodules on the mouse lung surface were photographed (**C**) and counted (**D**). H&E staining was performed to examine the pulmonary micro-metastatic foci in mice harboring FXR-silenced H1975 stable cells (**E**, **F**) or FXR-overexpressed A549 stable cells with Z-GS administration (**G**, **H**). Representative images (**E** and **G**; magnification, ×40; scale bar, 500 µm) and quantitative results (**F**, **H**) are shown. The pulmonary micro-metastatic foci were indicated by black arrows. IHC staining was conducted to examine the expression of FXR, IL-6, IL-6ST and p-STAT3 (Tyr705) in pulmonary metastatic tumors in mice harboring FXR-silenced H1975 stable cells (**I**) or FXR-overexpressed A549 stable cells with Z-GS administration (**J**). Representative images (magnification, ×200; scale bar, 50 µm) are shown. Data are shown as mean ± SD. n = 6 mice/group. ***p < 0.001, compared with the shNC or mock group. ^†††^*p* < 0.001, compared with A549-FXR group.

In this section, we first validated the pro-metastatic ability of FXR in NSCLC in vivo. H1975 cells with or without stable FXR knockdown (shFXR or shNC), or A549 cells with or without stable FXR overexpression (FXR or mock), were injected into the tail vein of nude mice to establish a mouse model of NSCLC metastasis. The gross metastatic nodules on the mouse lung surface, as well as the micro-metastatic foci inside the mouse lungs were counted. Consistent with the in vitro findings, the knockdown of FXR in H1975 significantly reduced lung surface metastasis in mouse NSCLC metastasis models (Fig. 5A, B). By contrast, the number of pulmonary metastatic nodules was significantly higher in FXR-overexpressed A549 cell-bearing mice, as compared with the mock group (Fig. 5C, D). H&E staining analysis of micro-metastatic foci in the lung tissues showed similar results (Fig. 5E–H). In parallel, IHC staining was performed to validate the regulation of IL-6, IL-6ST, and p-STAT3 (Tyr705) by FXR in vivo. The expression of IL-6, IL-6ST, and p-STAT3 (Tyr705) were significantly lower in FXR-silenced H1975 metastatic tumors than in corresponding control metastatic tumors (Fig. 5I). Conversely, metastatic lung tumors derived from FXR-overexpressed A549 cells exhibited significantly increased levels of IL-6, IL-6ST, and p-STAT3 (Tyr705) compared with those derived from mock A549 cells (Fig. 5J). These data confirmed that FXR promoted NSCLC metastasis in vivo.

Z-GS, a natural FXR inhibitor, has been shown to demonstrate anti-proliferative and anti-angiogenic effects in treating human colon and prostate cancer, respectively [26, 27]. Herein, the potential anti-metastatic activity of Z-GS was evaluated in this mouse NSCLC metastasis model in vivo. Mice harboring FXR-overexpressed A549 cells were treated with increasing doses of Z-GS every 3 days for 55 days. As shown in Fig. 5C, D, Z-GS treatment significantly inhibited lung surface metastasis in FXR-overexpressed A549 cell-bearing mice in a concentration-dependent manner. H&E staining showed a similar trend in that Z-GS dose-dependently reduced the number of lung micro-metastatic foci in this mouse NSCLC metastasis model (Fig. 5G, H). Moreover, Z-GS also decreased the expression of FXR, IL-6, IL-6ST, and p-STAT3 (Tyr705) in FXR-overexpressed A549 metastatic tumors (Fig. 5J). However, the average body weight did not differ between Z-GS-treated and control mice (Supplementary Fig. S3). The above results collectively suggested that this FXR inhibitor, Z-GS, decreased the FXR/IL-6/IL-6ST/p-STAT3 axis and reduced FXR^high^ NSCLC metastasis in mouse models in vivo.

### FXR is positively correlated with the expression of IL-6, IL-6ST and p-STAT3 (Tyr705), and is indicative of a poor prognosis in NSCLC

**Fig. 6:**
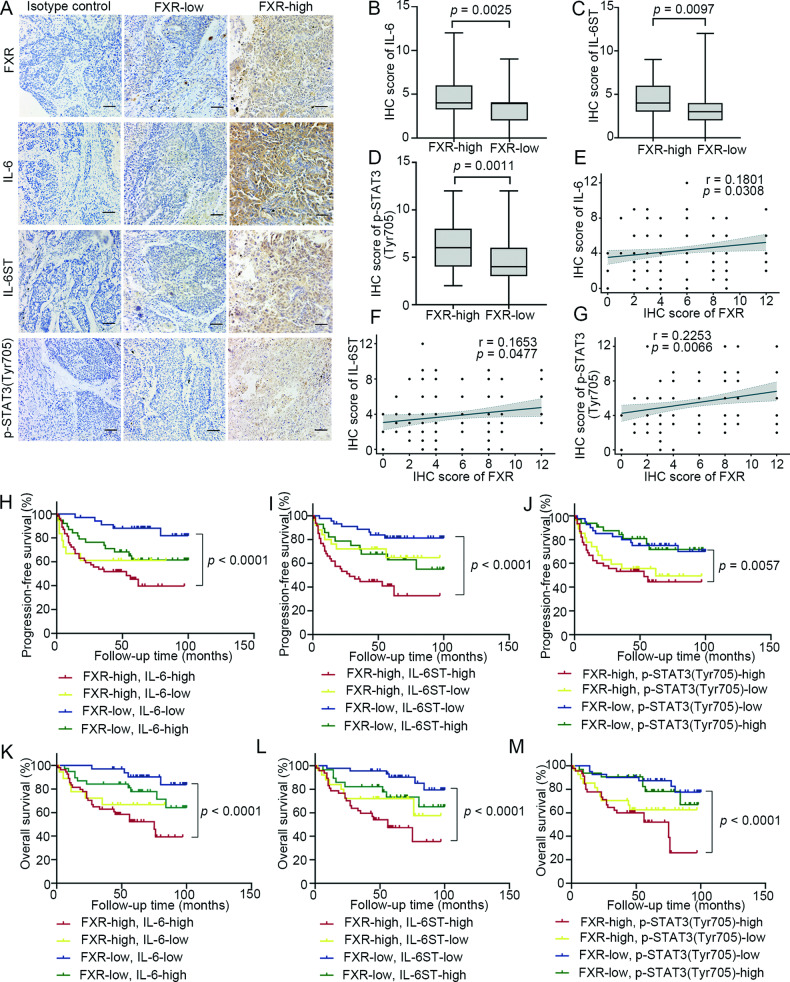
FXR is positively correlated with the expression of IL-6, IL-6ST and p-STAT3 (Tyr705), which indicate poor prognosis in NSCLC patients. A total of 144 clinical NSCLC samples were subjected to IHC staining. **A** Representative images showing the expresssion of FXR, IL-6, IL-6ST and p-STAT3 (Tyr705) in serial sections of “FXR high” NSCLC samples and “FXR low” NSCLC samples (Scale bar, 50 µm). Isotype control: the primary antibody was replaced by nonspecific mouse IgG. The IHC scores of IL-6 (**B**), IL-6ST (**C**) and p-STAT3 (Tyr705) (**D**) are higher in “FXR high” NSCLC (*n* = 72) than in “FXR low” NSCLC (*n* = 72) (Mann-Whitney *U* test). The line and box indicate the median and interquartile range. The bars indicate minimum and maximum values. Spearman’s correlation analysis indicated significant positive correlations between FXR and IL-6 expression (**E**), between FXR and IL-6ST expression (**F**), and between FXR and p-STAT3 (Tyr705) expression (**G**) in the entire cohort. Kaplan–Meier survival curves for PFS in NSCLC patients according to FXR and IL-6 levels (**H**; FXR^high^IL-6^high^ vs. FXR^low^IL-6^low^, *p* < 0.0001, log-rank test), according to FXR and IL-6ST levels (**I**; FXR^high^IL-6ST^high^ vs. FXR^low^IL-6ST^low^, *p* < 0.0001, log-rank test), or according to FXR and p-STAT3 (Tyr705) levels (**J**; FXR^high^p-STAT3^high^ vs. FXR^low^p-STAT3^low^, *p* = 0.0057, log-rank test). Kaplan–Meier survival curves for OS in NSCLC patients according to FXR and IL-6 levels (**K**; FXR^high^IL-6^high^ vs. FXR^low^IL-6^low^, *p* < 0.0001, log-rank test), according to FXR and IL-6ST levels (**L**; FXR^high^IL-6ST^high^ vs. FXR^low^IL-6ST^low^, *p* < 0.0001, log-rank test), or according to FXR and p-STAT3 (Tyr705) levels (**M**; FXR^high^p-STAT3^high^ vs. FXR^low^p-STAT3^low^, *p* < 0.0001, log-rank test).

Finally, we evaluated the relationship between the expression of FXR and IL-6, IL-6ST, and p-STAT3 (Tyr705) in clinical NSCLC samples. As shown in Fig. 6A–D, the expression levels of IL-6, IL-6ST and p-STAT3 (Tyr705) were significantly higher in the “FXR high” samples than in the “FXR low” samples. Moreover, Spearman’s correlation analysis revealed that the expression of FXR was positively correlated with the levels of IL-6, IL-6ST and p-STAT3 (Tyr705) in the clinical NSCLC cohort (Fig. 6E–G). Then, NSCLC samples were stratified according to FXR, IL-6, IL-6ST and p-STAT3 (Tyr705) levels. Survival analysis indicated that patients with both “FXR high” and “IL-6 high” (characterized by FXR^high^IL-6^high^) NSCLC, FXR^high^IL-6ST^high^ NSCLC, or FXR^high^p-STAT3^high^ NSCLC had a significantly worse progression-free survival (PFS) as compared with FXR^low^IL-6^low^, FXR^low^IL-6ST^low^, and FXR^low^p-STAT3^low^ NSCLC patients, respectively (Fig. 6H–J). Additionally, patients with FXR^high^IL-6^high^, FXR^high^IL-6ST^high^, or FXR^high^p-STAT3^high^ NSCLC had a significantly shorter overall survival (OS) than FXR^low^IL-6^low^, FXR^low^IL-6ST^low^, and FXR^low^p-STAT3^low^ NSCLC patients, respectively (Fig. 6K–M). Taken together, the above findings demonstrated that FXR is positively correlated with the expression of IL-6, IL-6ST and p-STAT3 (Tyr705), and this is indicative of a poor prognosis in NSCLC patients.

To further validate our findings, data obtained from TCGA was analyzed. There was no information regarding p-STAT3 mRNA expression in the data obtained from TCGA. Despite this, there was a slight positive correlation between *NR1H4* (a gene that encodes FXR) and *STAT3* mRNA expression in a dataset of 994 NSCLC samples (Supplementary Fig. S4A). Notably, *NR1H4* mRNA levels were positively correlated with *IL-6ST* mRNA levels in this dataset (Supplementary Fig. S4B). However, there was no correlation between *NR1H4* and *IL-6* mRNA expression levels in NSCLC in the data obtained from TCGA (Supplementary Fig. S4C). Survival analysis showed that FXR^high^STAT3^high^ NSCLC had a significantly worse OS compared with the FXR^low^STAT3^low^ subgroup (Supplementary Fig. S4D). Additionally, a worse, but not significant OS was observed in the FXR^high^IL-6ST^high^ NSCLC patients than in FXR^low^IL-6ST^low^ NSCLC patients (Supplementary Fig. S4E).

## Discussion

**Fig. 7:**
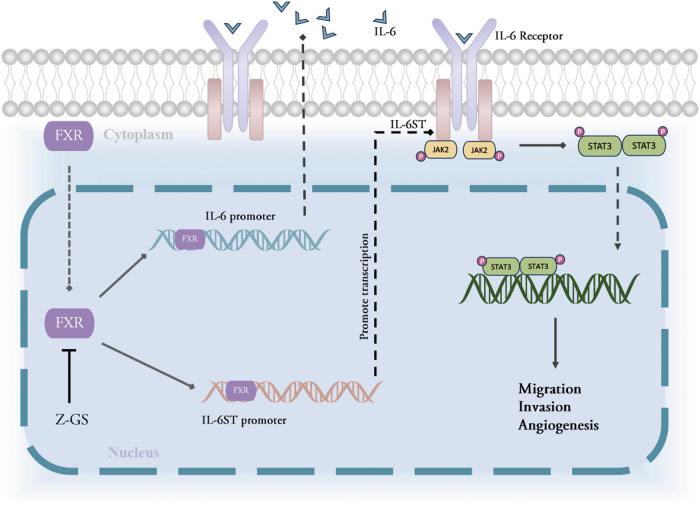
Schematic illustration of the mechanism by which FXR promotes NSCLC metastasis. In NSCLC cells, FXR binds specifically to the promoters of *IL-6ST* and *IL-6* genes to upregulate their transcription, which leads to increased activation of the Jak2/STAT3 signaling pathway. The activated Jak2/STAT3 signaling regulates downstream target genes to promote NSCLC cell migration, invasion, and angiogenesis. The FXR inhibitor Z-GS can decrease FXR/IL-6/IL-6ST/p-STAT3 axis and reduce FXR^high^ NSCLC metastasis.

Metastasis accounts for more than 90% of cancer-related deaths in NSCLC [28]; however, options for treating metastatic NSCLC are currently limited. Thus, the elucidation of the biological underpinnings that govern metastatic processes should facilitate the development of novel therapeutic agents or strategies for metastatic NSCLC. We previously reported that FXR promotes NSCLC tumor growth via upregulating *CCND1* transcription and remodeling an immunosuppressive microenvironment [18, 20]. This represents the first systematic study to evaluate the function of FXR in NSCLC metastasis. We found that FXR promotes the migration, invasion, and angiogenic ability of NSCLC cells in vitro, and increases NSCLC metastasis in mouse models in vivo. Mechanistically, FXR exerts its pro-metastatic effect by upregulating IL-6ST and IL-6 via direct transactivation, thereby leading to activation of the Jak2/STAT3 signaling pathway in NSCLC cells (Fig. 7). These findings are supported by data from clinical NSCLC samples and data from TCGA, showing that FXR positively correlates with the expression of IL-6, IL-6ST and p-STAT3 in NSCLC tissues, and this is also more indicative of a poor prognosis. Of note, pharmaceutical inhibition of FXR significantly suppresses IL-6/IL-6ST/p-STAT3 signaling and reduces metastatic tumor burden in mouse NSCLC metastasis models, suggesting a potential therapeutic approach for this subgroup (characterized by FXR^high^) of metastatic NSCLC patients (Fig. 7).

FXR is an adopted nuclear receptor that is essential for regulating bile acid, cholesterol, glucose, and lipid homeostasis [12, 13]. However, emerging evidence has implicated the deregulation of FXR in several malignancies. While FXR deficiency in mice was linked to the development of spontaneous liver and intestinal tumors [14, 16], others have described the oncogenic activities of FXR in human breast, esophageal, pancreatic, and clear-cell renal cancers [15, 17, 29, 30]. In addition to the previously reported role in promoting local tumor growth [18, 20], the present study highlights the biological functions of FXR in promoting NSCLC metastasis. We showed that FXR promotes NSCLC cell migration, invasion, and angiogenesis in vitro, and NSCLC metastasis in mouse models in vivo, collectively suggesting a comprehensive oncogenic role for FXR in NSCLC development and progression. Distinct environmental cues and gene expression profiles are proposed to determine the tissue-specific actions of FXR in tumorigenesis. In line with this interpretation, FXR was found to promote tumor migration and invasion in bile acid-deprived pancreatic and clear-cell renal cancer [15, 30], consistent with our findings in NSCLC, but exerts an opposite effect in tumor metastasis in bile acid-enriched hepatocellular and colon carcinomas [31, 32].

Aberrant STAT3 activation was detected in over 60% of NSCLC patients [33], which plays oncogenic roles in cell survival, stemness, metastasis, angiogenesis, drug resistance, and immune evasion [6, 8]. This study demonstrated that FXR activated the STAT3 signaling pathway, which reciprocally mediated the promoting effect of FXR in NSCLC migration, invasion, and angiogenesis. Consistently, the FXR agonist GW4064 was reported to increase STAT3 activation in endometriotic stromal cells, whereas FXR inhibitor Z-GS treatment suppressed both STAT3 phosphorylation and activities in multiple myeloma cells [34, 35]. STAT3 signaling is under the control of receptor or non-receptor tyrosine kinases, cytokines, and growth factors hormones [36]. Specifically, STAT3 can be activated by the canonical IL-6 via binding to the IL-6Rɑ/IL-6ST complex and recruiting Jak1/2 kinases [7]. IL-6 expression was reported to be increased in NSCLC serum and correlated with distant metastasis [37, 38]. Herein, IL-6 was found to be increased in the culture supernatant of FXR-overexpressed NSCLC cells. Moreover, we found that FXR upregulates IL-6 and IL-6ST expression but not IL6Rɑ, thus suggesting that IL-6/IL-6ST may mediate FXR-modulated Jak2/STAT3 activation and tumor metastasis in NSCLC. In support of this hypothesis, IL-6ST knockdown largely abrogated the promoting effects of FXR on Jak2/STAT3 signal activation and the following migration, invasion, and angiogenesis in NSCLC. As a transcription factor, FXR controls the downstream target gene via binding the FXRE sequences in the promoter region, either as a classical FXR-RXRɑ heterodimer or as a monomer [25]. This study provides compelling and novel evidence that FXR upregulates IL-6/IL-6ST in NSCLC via direct binding to the predicted FXRE motifs in *IL-6ST* and *IL-6* promoters, respectively. The above findings, which collectively demonstrated that FXR induces metastasis in NSCLC by transactivating IL-6/IL-6ST and triggering the downstream Jak2/STAT3 signaling cascades, are of particular significance. Since IL-6 mediates a variety of biological activities, such as B-cell differentiation and T-cell proliferation [39], targeting IL-6/Jak2/STAT3 signals using an IL-6 neutralizing antibody may face challenges due to the systemic off-target toxicities [40]. In comparison, it is more plausible to target the upregulated FXR in NSCLC that specifically induces the excessive or pathogenic IL-6/Jak2/STAT3 signaling pathway on the matter of tumor metastasis. In agreement with this notion, we observed positive correlations between FXR expression and IL-6, IL-6ST, and p-STAT3 (Tyr705) expression in NSCLC from both clinical samples and data obtained from TCGA, and this was indicative of a poor prognosis.

How the FXR-driven metastasis in NSCLC can be treated in vivo is of particular interest. Z-GS is a phytosterol derived from the resin of the *Commiphora mukul* tree, which has long been used in *Ayurvedic* medicine to treat hyperlipidemia, obesity, hypothyroidism, and arthritis [41]. Recently, emerging evidence has documented the anti-tumor properties of Z-GS in several tumors, including prostate, colorectal, and pancreatic cancer [26, 42, 43]. It is widely acknowledged that Z-GS works as an inhibitor for FXR [22]. We previously reported that Z-GS exerts a potent anti-proliferation effect, which leads to undesirable PD-L1 upregulation via blocking FXR in NSCLC cells [18, 21]. Herein, Z-GS was utilized for pharmacologic inhibition of FXR for treating metastatic NSCLC. Our results showed that Z-GS dose-dependently inhibited FXR-induced NSCLC migration, invasion, and angiogenesis in vitro, and reduced FXR^high^ NSCLC metastasis in mouse models in vivo. IHC analysis also revealed a synchronous reduction of FXR and downstream targets, namely, IL-6, IL-6ST, and p-STAT3 (Tyr705), in Z-GS-treated NSCLC tumors in vivo. Consistent with our data, Z-GS was found to inhibit angiogenesis, and exert anti-metastatic effects via inhibition of the Jak/STAT pathway in prostate and pancreatic cancers [27, 43]. Based on the present study, the FXR inhibitor Z-GS should be a promising anti-metastatic agent for this subgroup of FXR^high^ NSCLC patients. Future pre-clinical and clinical studies are needed to validate this therapeutic strategy.

In conclusion, the present study demonstrated that FXR promotes NSCLC metastasis through transcriptionally upregulating IL-6ST and IL-6 and activating the downstream Jak2/STAT3 signals. FXR inhibitor, Z-GS, showed a favorable therapeutic effect in decreasing FXR/IL-6/IL-6ST/p-STAT3 axis and treating FXR^high^ NSCLC metastasis in mouse models in vivo. These findings strongly suggest FXR as a prognostic biomarker and therapeutic target for NSCLC metastasis.

### Reporting summary

Further information on research design is available in the Nature Research Reporting Summary linked to this article.

### Supplementary information

Supplementary Materials

Supplementary Table S1

Supplementary Figure S1

Supplementary Figure S2

Supplementary Figure S3

Supplementary Figure S4

Original data

Reporting Summary

## Data Availability

The *NR1H4, STAT3, IL-6ST* and *IL-6* mRNA expression data and survival data in NSCLC generated in this study are publicly available in The Cancer Genome Atlas (TCGA) at https://portal.gdc.cancer.gov/. The TCGA accession codes were listed in Supplementary Table S1. Other data generated or analyzed in this study are available from the corresponding author on reasonable request.
