# Supplementary material for: Farnesoid X receptor promotes non-small cell lung cancer metastasis by activating Jak2/STAT3 signaling via transactivation of IL-6ST and IL-6 genes
Source: Cell Death Dis. 2024 Feb 15;15(2):148. doi: 10.1038/s41419-024-06495-y (PMC10869786; doi:10.1038/s41419-024-06495-y)
Supplement: Supplementary file 1 — Supplementary Materials [file 41419_2024_6495_MOESM1_ESM.doc]

**Farnesoid X receptor promotes non-small cell lung cancer metastasis by activating Jak2/STAT3 signaling via transactivation of IL-6ST and IL-6 genes**

Xiuye Jin^1,2,5,6,9,#^, Bin Shang^3,4,5,6,#^, Junren Wang^1,5,6^, Jian Sun^1,2,5,6^, Jing Li^7^, Bin Liang^1,2,5,6^, Xingguang Wang^1,2,5,6^, Lili Su^1,2,5,6^, Wenjie You^1,2,5,6,8^,* and Shujuan Jiang^1,2,5,6^,*

**Supplementary figure legends**

**Supplementary Figure S1.**

The effects of FXR on IL-6Rɑ expression and YAP and Notch signaling pathways in NSCLC cells. (A-B) Western blotting was performed to evaluate the protein levels of IL-6Rɑ in FXR-silenced A549 and H1975 cells (A) and FXR-overexpressed A549 stable cells treated with increasing concentrations of Z-GS (0, 10, 20, and 40 µM) (B). GAPDH was used as a loading control. (C-D) qRT-PCR was performed to examine the mRNA levels of IL-6Rɑ in FXR-silenced A549 and H1975 cells (C) and FXR-overexpressed A549 stable cells treated with increasing concentrations of Z-GS (0, 10, 20, and 40 µM) (D). β-actin served as an internal control. (E-F) Western blotting was performed to evaluate the protein levels of cleaved Notch1, p-YAP1 (Tyr357), YAP, and HES1 in FXR-silenced A549 and H1975 cells (E) and FXR-overexpressed A549 stable cells (F). GAPDH was used as a loading control. Data represents mean ± SD from at least three independent experiments. ** *p* < 0.01, compared with the NC group. † *p* < 0.05, compared with A549-FXR group. NS, not significant.

**Supplementary Figure S2.**

FXR specifically binds to the second putative FXRE motif in *IL-6ST* promoter and the first-to-third putative FXRE motifs in *IL-6* promoter. (A) ChIP assays were performed in H1975 and A549 cells using anti-human FXR/NR1H4 antibody and primer corresponding to the first (upper bands), second (middle bands), or third (lower bands) putative FXRE motif in *IL-6ST* promoter. (B) ChIP assays were performed in H1975 and A549 cells using anti-human FXR/NR1H4 antibody and primer corresponding to the first-to-third (top bands), fourth (middle upper bands), fifth (middle lower bands), or sixth (bottom bands) putative FXRE motif in *IL-6* promoter. Representative PCR amplification products are shown. Chromatin obtained with isotype IgG and non-immunoprecipitated samples (input) served as negative and positive controls, respectively.

**Supplementary Figure S3.**

FXR increases NSCLC metastasis *in vivo*, which is reduced by FXR inhibitor Z-GS. A mouse model of NSCLC metastasis was established as described in Figure 5. The body weight of mice harboring FXR-silenced H1975 stable cells (A) or FXR-overexpressed A549 stable cells with Z-GS administration (B) were monitored every 3 days. Data are shown as mean ± SD. n = 6 mice/group.

**Supplementary Figure S4.**

Correlation analysis between FXR and IL-6, IL-6ST, and STAT3 in NSCLC of TCGA cohort. (A-C) Spearman’s rank test was conducted to evaluate the correlations between FXR and STAT3 (A), between FXR and IL-6ST (B), and between FXR and IL-6 (C) in 994 NSCLC from TCGA datasets. (D) Kaplan-Meier overall survival curves of FXR^high^STAT3^high^, FXR^high^STAT3^low^, FXR^low^STAT3^high^ and FXR^low^STAT3^low^ NSCLC from TCGA (FXR^high^STAT3^high^ vs. FXR^low^STAT3^low^, *p* = 0.0365, log-rank test). (E) Kaplan-Meier overall survival curves of FXR^high^IL-6ST^high^, FXR^high^IL-6ST^low^, FXR^low^IL-6ST^high^ and FXR^low^IL-6ST^low^ NSCLC from TCGA (FXR^high^IL-6ST^high^ vs. FXR^low^IL-6ST^low^, *p* = 0.1263, log-rank test).
