# Supplementary figures and images for: Farnesoid X receptor promotes non-small cell lung cancer metastasis by activating Jak2/STAT3 signaling via transactivation of IL-6ST and IL-6 genes
Source: Cell Death Dis. 2024 Feb 15;15(2):148. doi: 10.1038/s41419-024-06495-y (PMC10869786; doi:10.1038/s41419-024-06495-y)

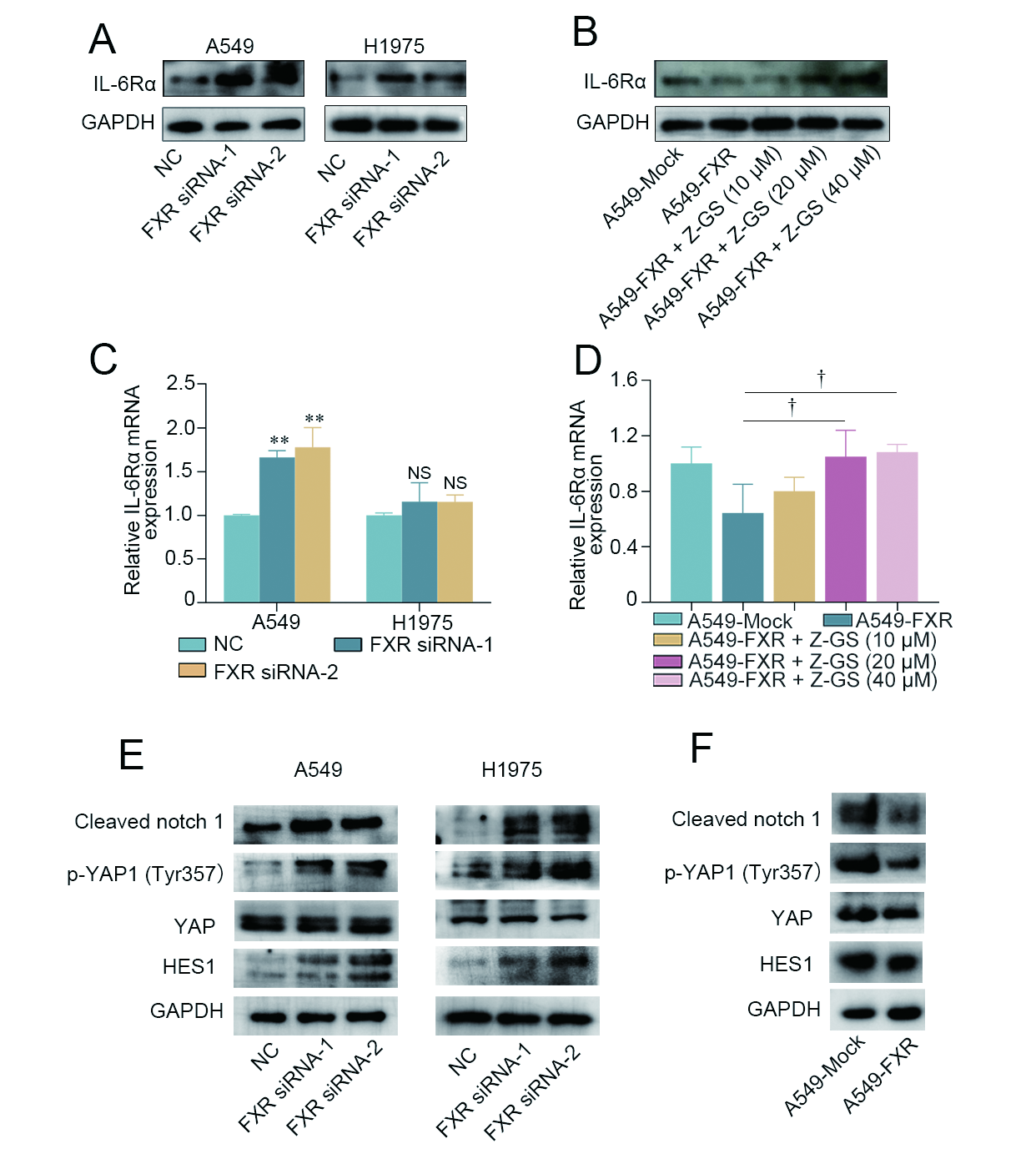

Supplement: Supplementary file 3 — Supplementary Figure S1 [file 41419_2024_6495_MOESM3_ESM.tif]

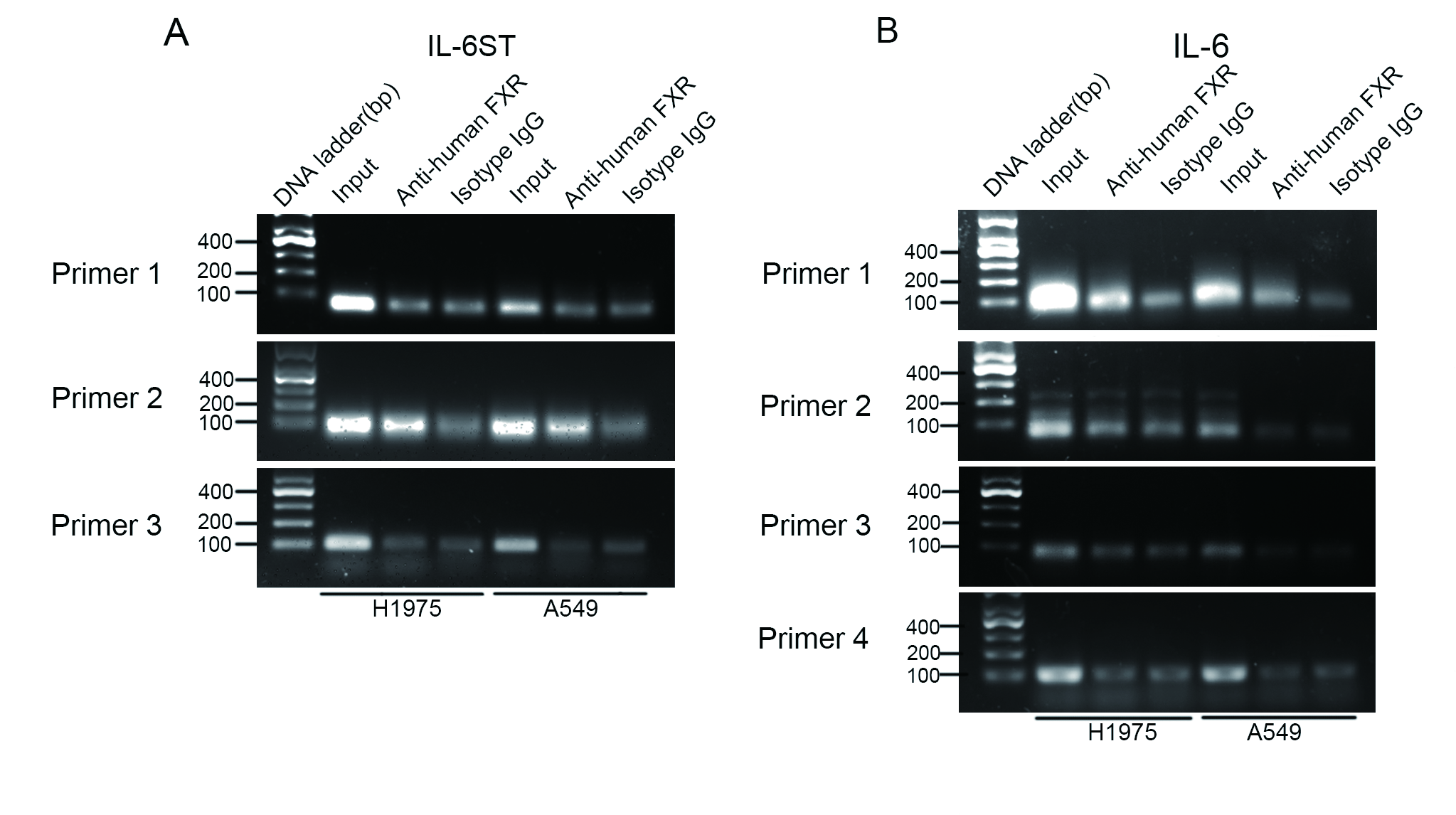

Supplement: Supplementary file 4 — Supplementary Figure S2 [file 41419_2024_6495_MOESM4_ESM.tif]

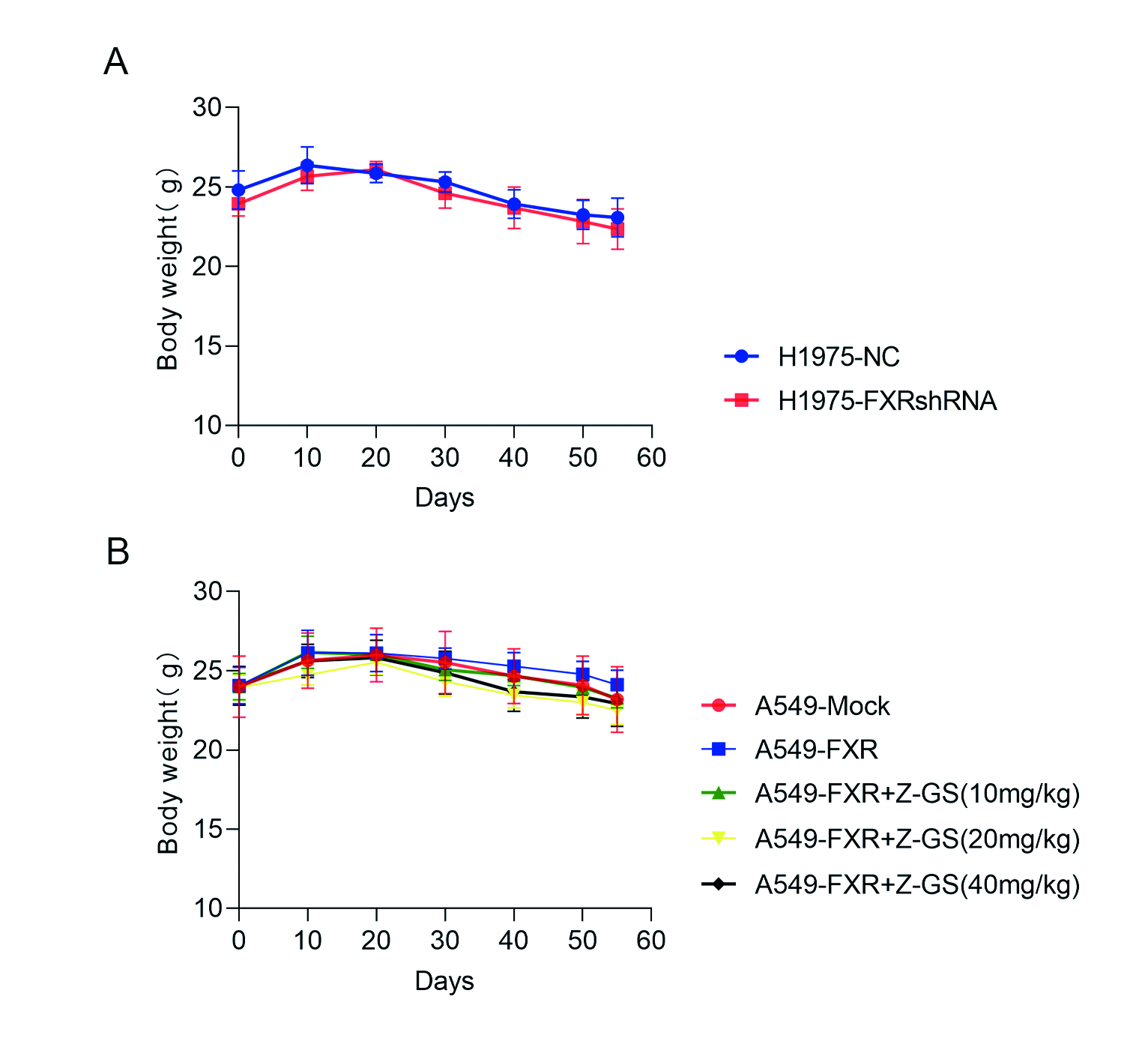

Supplement: Supplementary file 5 — Supplementary Figure S3 [file 41419_2024_6495_MOESM5_ESM.tif]

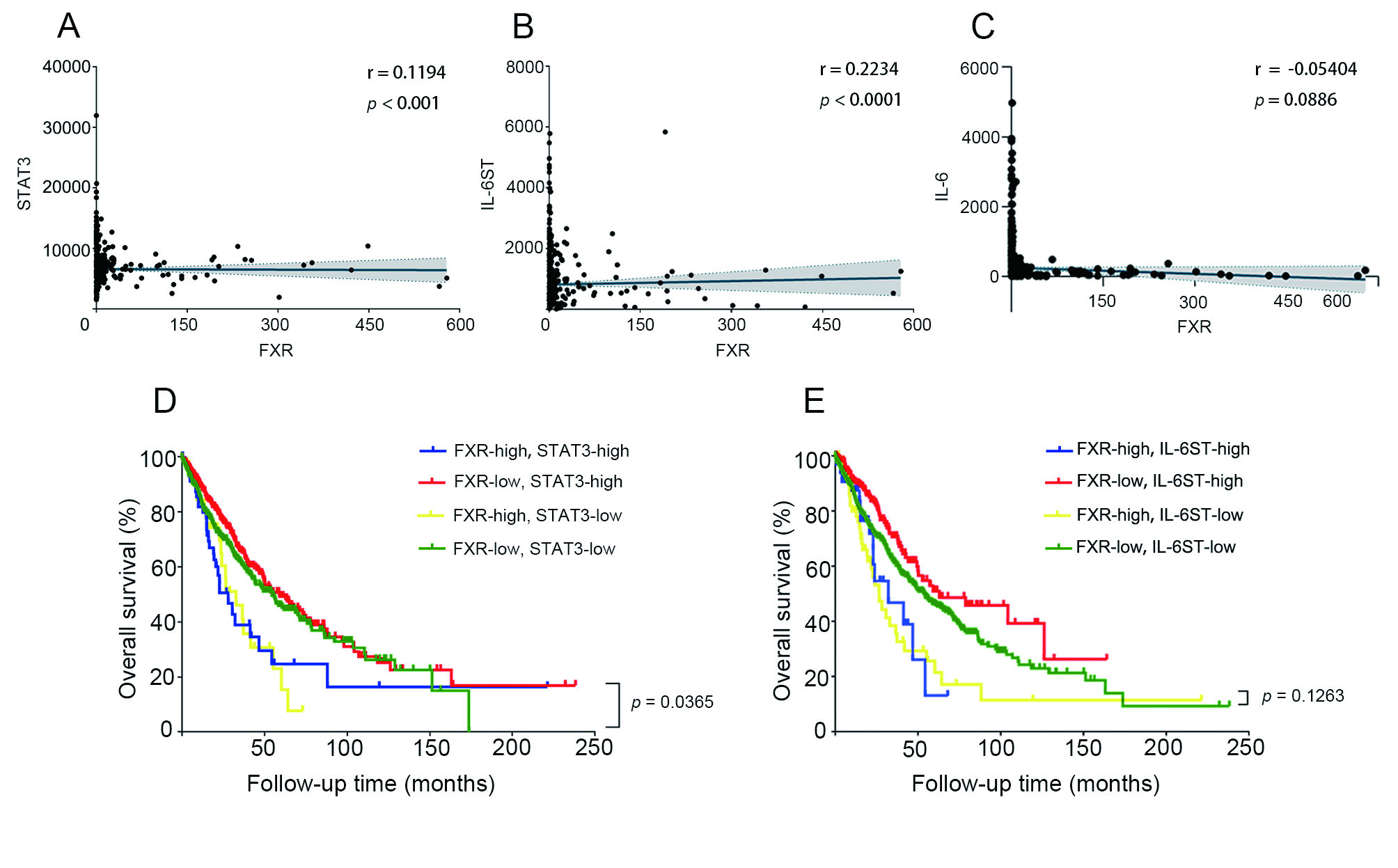

Supplement: Supplementary file 6 — Supplementary Figure S4 [file 41419_2024_6495_MOESM6_ESM.tif]

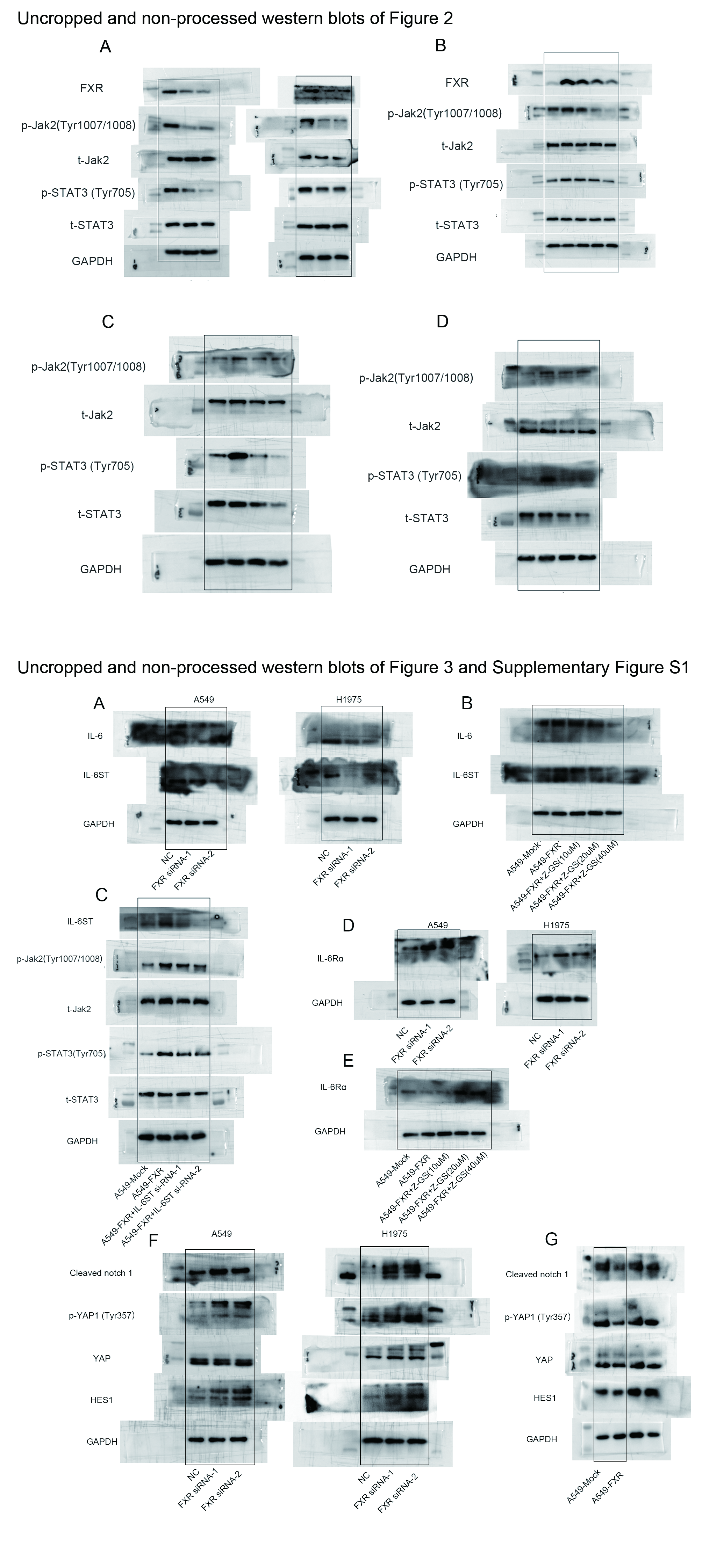

Supplement: Supplementary file 7 — Original data [file 41419_2024_6495_MOESM7_ESM.tif]
